# Young people in HIV care in Ukraine: a national survey on characteristics and service provision

**DOI:** 10.12688/f1000research.18573.2

**Published:** 2019-05-14

**Authors:** Galyna Kyselyova, Violeta Martsynovska, Alla Volokha, Nataliya Nizova, Ruslan Malyuta, Ali Judd, Claire Thorne, Heather Bailey

**Affiliations:** 1 Shupyk National Medical Academy of Postgraduate Education, Kiev, Ukraine; 2 The Public Health Center of the Ministry of Health of Ukraine, Kiev, Ukraine; 3 Institute of Epidemiology and Infectious Diseases of NAMS, Kiev, Ukraine; 4 Perinatal Prevention of AIDS Initiative, Odessa, Ukraine; 5 MRC Clinical Trials Unit at UCL, Institute of Clinical Trials and Methodology, University College London, London, UK; 6 Population, Policy and Practice Programme, UCL Great Ormond Street Institute of Child Health, University College London, London, UK

**Keywords:** HIV, youth, adolescents, Ukraine, Eastern Europe, transition, perinatal HIV infection, injecting drug use, reproductive health, harm reduction

## Abstract

**Background: **Ukraine’s perinatally HIV-infected (PHIV) young people are ageing into adolescence/young adulthood and, alongside those with horizontally-acquired HIV infections, require transitional and other support services. We aimed to map this population and policies/service provision at specialist HIV centres, to inform future service development.

**Methods: **A national survey was conducted of 28 HIV/AIDS centres on number, characteristics (age group, HIV acquisition mode) and care setting (paediatric/adult) of 10-24 year olds in HIV care in each of 24 regions in January 2016. Information was collected on policies/service provision at each centre.

**Results: **Of 13,286 young people aged 10-24 years registered for HIV care nationally in Ukraine in January 2016, 1,675 were aged 10-18 years. Three-quarters of ≤19 year olds were PHIV, while 72% of 20-24-year-olds had sexually-acquired infection. Five regions accounted for two-thirds of 10-18 year olds in paediatric and 85% of 19-24 year olds in adult services.

In 2015, 97 young people transitioned from paediatric to adult services nationally, typically at 18 years although with flexibility in timing at 17/28 centres. At 27/28 centres, horizontally HIV-infected young people aged <18 years began their HIV care in paediatric services sometimes (5) or always (22). Transition support most commonly consisted of a joint appointment with paediatrician and adult doctor, and support from a psychologist/social worker (both at 24/28 centres).

Only 5/28 centres offered routine HIV care during the evening or weekend, and availability of integrated sexual/reproductive health and harm reduction services was uneven. Of 16/28 centres selectively following-up patients who did not attend for care, 15 targeted patients in paediatric services.

**Conclusions: **Heterogeneity in the population and in service availability at the main regional/municipal HIV/AIDS centres has implications for potential structural barriers to HIV care, and development of services for this group.

## Abbreviations

ART, antiretroviral therapy; IDU, injecting drug use; PHIV, perinatally HIV-infected; PLHIV, people living with HIV; PWID, people who inject drugs

## Introduction

Ukraine, a middle income country, has the second largest population of people living with HIV (PLHIV) in Europe, estimated at 238,000 ^1^. Important successes in prevention of mother-to-child transmission and paediatric treatment programmes are reflected, as in other settings, in the demographic characteristics of perinatally HIV-infected (PHIV) patients, who are an ageing cohort. National data indicate that there were 3,014 HIV-infected children and young people aged <18 years in HIV care in Ukraine at the end of 2016 ^1^. Around half of the PHIV population were estimated to be aged ≥10 years at this time with numbers transferring to adult care expected to peak in 2019–2026, based on an age at transfer of around 18 years ^2^.

In terms of horizontally-acquired HIV injecting drug use (IDU) has been a key driver of the epidemic among young people in Ukraine ^3^, including among marginalised youth ^4^ as average age of IDU initiation is in the late teens ^5^. In recent years, young people aged 15–24 years have accounted for a declining number and proportion of newly diagnosed HIV infections (12% in 2009 vs. 5.2% in 2016) ^1^. However, an estimated 42% of PLHIV in Ukraine are undiagnosed ^6^, and this proportion may be higher among young people, reflecting the specific barriers to services among young people who inject drugs (PWID) and their sexual partners ^7^. Women accounted for 43% of new infections in 2015, but with a younger age of infection/diagnosis than men; a third of 15–49 year old women living with HIV were <30 years in 2015 vs. only 19% of men ^8^.

HIV services in Ukraine are delivered through specialist regional and municipal HIV/AIDS centres, in conjunction with local or satellite clinics to which antiretroviral therapy (ART) provision has been decentralised in recent years. Decentralised services are available mainly to adults, while children usually remain under the follow-up of a paediatrician at a specialist centre (personal communication, Galyna Kyselyova). HIV treatment and care are officially provided free-of-charge, however a system of unofficial payments may also apply, and support services are often provided by non-governmental organisations. Around two-thirds of diagnosed individuals linked to care were on ART in 2016 ^1^.

Adolescence and young adulthood are periods of vulnerability regarding access to and retention in healthcare services for chronic conditions, including HIV, due to a range of factors including increased risk-taking behaviours, challenges around transferring to and navigating adult-oriented health systems, managing stigma and disclosure, and emergence of mental health problems ^9, 10^. Findings from high income settings have indicated that PHIV young people are at elevated risk of poor ART adherence, virological failure and deterioration of health and loss to follow-up during adolescence and transition to adult care ^11– 14^, although UK data show substantial improvements over calendar time and improved CD4 trajectory in some groups post-transition ^15, 16^. The few studies to date evaluating different service delivery models for HIV-positive young people indicate the importance of clinic accessibility, integrated care and peer support, as well as the potential impact of individual-level interventions such as financial incentives for clinic attendance and treatment adherence ^17, 18^. However, quality of evidence regarding models of care for young people is poor, and the success of different models is likely to be highly setting-specific, while most studies are from high income settings.

Reforms of Ukraine’s healthcare system are ongoing and include development of more patient-centred, outcome-oriented models of care ^19^. However, currently there is little evidence to guide the development of policies, care and support programmes specific to young PLHIV, despite evolving needs for services. The objectives of this study were to describe the contemporary population of young people aged 10–24 years receiving HIV care in Ukraine by mode of HIV acquisition, type of care (paediatric vs. adult) and region, and to document current service provision and local policies at specialist HIV centres in order to inform future service development.

## Methods

In April and May 2016, a paper-based questionnaire was sent to 24 regional HIV/AIDS centres and four large municipal HIV/AIDS centres (Kryvy Rih and Dnipro city centres in Dnepropetrovsk region and Bila Tserkva and Kiev city centres in Kiev region) in collaboration with the Public Health Center of the Ministry of Health of Ukraine. These 28 centres were surveyed because they collate data on all people registered for HIV care nationally, within each of the 24 regions (oblasts), including those followed-up at local or satellite clinics.

The questionnaire requested the number of 10–24 year olds (young people, according to WHO’s definition ^20^) receiving HIV care within the region on 1 January 2016. Based on an age of transfer to adult care of 18 years, the questionnaire requested the number of young people in paediatric and adult services in two age groups: 10–18 years and 19–24 years. To explore service needs in more depth, the number of young people by mode of HIV acquisition was disaggregated according to three age groups: 10–14 years, 15–19 years and 20–24 years, to accommodate WHO definition of adolescence (10–19 years) ^20^ and facilitate comparison with national figures. Additional questions were on policies around transition, provision of psychological support, sexual and reproductive health and harm reduction services and practices around loss to follow-up at each regional/municipal centre. Responses were provided by a paediatrician at each centre or another member of staff with knowledge of models of care provided to young people.

To obtain an estimate of the total number of young people aged 10–24 years in HIV care nationally, we summed the regional totals. This national estimate was complete with the exception of the temporarily uncontrolled territories of the Donetsk and Lugansk regions (partial data available) and the Russian-occupied territories of The Crimea. To identify possible double-counting of young people registered simultaneously in paediatric and adult services within the same region, we compared the total across both services with the regional total by mode of HIV acquisition. Where the second total was smaller, we summed the 10–18 year olds registered in adult services and the 19–24 year olds registered in paediatric services, to reach an estimate of the maximum number double-counted across paediatric and adult services.

Data were entered into a REDCap database. Descriptive analyses were conducted in STATA version 13 (Stata Corp LP, College Station USA).

As a service evaluation, this survey did not require ethics approval, but was approved by the Ministry of Health. Individual patient data were not collected and consent was therefore not required.

## Results

All 28 centres responded; questionnaires were completed by paediatric/adult infectious diseases doctors ( *n*=23), epidemiologists ( *n*=2), an HIV/AIDS centre head ( *n*=1), immunologist ( *n*=1), and nurse ( *n*=1).

### Number of young people in HIV care nationally

**Figure 1.**
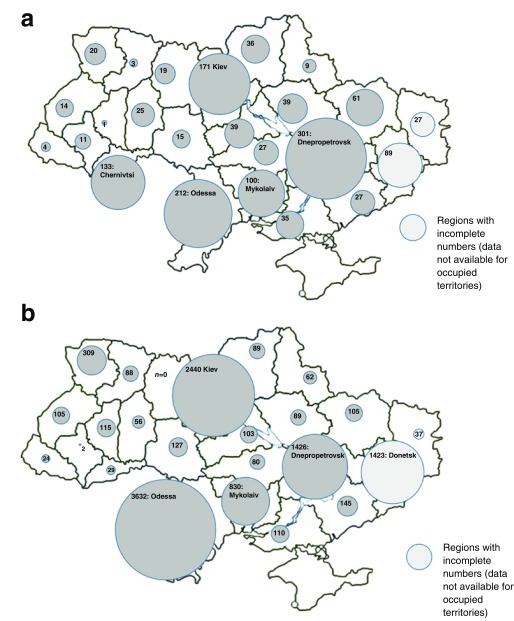
Maps showing ( **a**) number of 10–18 year olds in paediatric HIV care and ( **b**) number of 19–24 year olds in adult HIV care by region, on 1 January 2016. Area of circle is proportional to number of young people. Note different scales for maps ( **a**) and ( **b**). Name of region was included for centres with ( **a**) n≥100 10–18 year olds in paediatric services and ( **b**) n≥800 19–24 year olds in adult services. Maps were adapted from original versions created by Aleksandr Grigoryev, available at https://commons.wikimedia.org/w/index.php?curid=28880433 under a CC0 license.

Nationally, a total of 13,286 young people aged 10–24 years were registered for HIV care (paediatric and adult services) in January 2016, of whom 43 may have been registered simultaneously in both paediatric and adult services in the same region. Of the total 13,286, 13% (1,675) were aged 10–18 years, a group making up 94% (1418/1505) of those in paediatric services and 2% (257/11781) of those in adult services. The median number of 10–18 year olds registered in paediatric services in each region was 31 (IQR 17, 73; range 1, 212) while that for 19–24 year olds in adult care was 101 (IQR 59, 227; range 0, 3632). Dnepropetrovsk, Kiev, Mykolaiv and Odessa regions had amongst the largest numbers of patients in both age groups; along with Chernivtski, these regions accounted for almost two-thirds (65%, 917/1418) of 10–18 year olds in paediatric care ( Figure 1a) and with Donetsk they accounted for 85% (9849/11,524) of 19–24 year olds in adult HIV services ( Figure 1b). Overall, 839 10–24 year olds were newly registered for HIV care in 2015, 759 of whom were aged 19–24 years.

Table 1 shows mode of HIV acquisition of young people in HIV care by age group. Overall 13% (1663/13060) were adolescents (10–19 years), most of whom were perinatally-infected. There were 210 PHIV young people aged 20–24 years in HIV care at the beginning of 2016, accounting for 1.8% of this age group and 14% of the PHIV population overall. Among 15–19 year olds, 43% (294/691) had sexually-acquired HIV infection, increasing to 72% (8183/11397) in those aged 20–24 years. Young PWID accounted for a quarter of the 20–24 year old age group.

**Table 1.** Number of young people in HIV care in Ukraine by age group and mode of HIV acquisition.

Age group	Perinatal	Sexual	Injecting drug use	Other	Total
10–14 years	968 (99.5%)	2 (0.2%)	0	2 (0.2%)	972 (100%)
15–19 years	318 (46.0%)	294 (42.5%)	76 (10.9%)	3 (0.4%)	691 (100%)
20–24 years	210 (1.8%)	8183 (71.8%)	3001 (26.3%)	3 (0.03%)	11397 (100%)
**Total**	**1496 (11%)**	**8479 (65%)**	**3077 (24%)**	**8 (0.1%)**	**13060 ^†^ (100%)**

†This total is 226 fewer than the total 13286 given in text due to unknown /unreported mode of HIV acquisition for some young people, and potential double-counting of up to 43 patients registered with both paediatric and adult services simultaneously.

### Policies regarding transition from paediatric to adult HIV care

In total 97 patients transitioned from paediatric to adult care across 13 regions during 2015; 66 in two regions (Dnepropetrovsk and Odessa). In 11/28 centres, transition always took place at 18 years, in two centres it could sometimes be postponed depending on the paediatric caseload, while in 15 centres transition could always be postponed if necessary; of the latter group of 15 centres, there was no formal age limit for transfer in eight, while the maximum age limit ranged up to 24 years in the remainder. At the 17 centres with flexible policies, possible reasons for postponement of transfer included lack of maturity or independence of young person ( *n*=15), young person’s request ( *n*=11), poor support at home ( *n*=6), poor adherence ( *n*=3), cognitive problems ( *n*=3), poor health ( *n*=1), and financial concerns related to payment for tests or drugs in adult services ( *n*=1).

Most (22/28) centres reported that young people with horizontally-acquired HIV aged <18 years began their HIV care in paediatric services while in five centres they sometimes initiated care in adult services depending on factors such as adequate maturity, pregnancy, wish to attend adult services, or availability of a paediatrician; one centre reported always registering young people with horizontally-acquired HIV in adult HIV services.

Table 2 shows the number of centres offering each of four aspects of transition support. Most (24/28) centres offered young people a joint appointment with paediatrician and adult doctor as part of the transition process, with specific support from a psychologist or social worker also available at most centres surveyed. Only three centres reported that young people continued to see a paediatrician for a period after transfer to adult care.

**Table 2.** Number and proportion of regional/municipal HIV/AIDS centres offering different types of transition support.

	Overall ^†^	10 centres with no transfers in 2015	17 centres transferring ≥1 patient to adult care in 2015
	N (%) of centres	N (%) of centres	N (%) of centres
**Young person meets adult doctor before transfer**	14/27 (52%)	4/9 (44%)	9/17 (53%)
**Young person has appointment(s) with** **paediatrician and adult doctor together**	24/28 (86%)	9/10 (90%)	14/17 (82%)
**Young person has specific support with transfer** **from psychologist or social worker**	24/28 (86%)	9/10 (90%)	14/17 (82%)
**Young person continues to see paediatrician for** **a period after transfer to adult care**	3/25 (12%)	1/9 (11%)	2/15 (13%)

† One centre was missing data on transfers in 2015 and is included in the “overall” column only

### Support services and follow-up of young people

Of the 28 centres, five centres offered a weekend or evening clinic (for four this was at least weekly) while two centres reported out-of-hours services for emergencies only (e.g. post-exposure prophylaxis). All centres provided a “walk-in” service without an appointment during opening hours, either for standard follow-up ( *n*=27) or only for urgent care ( *n*=1).

**Figure 2.**
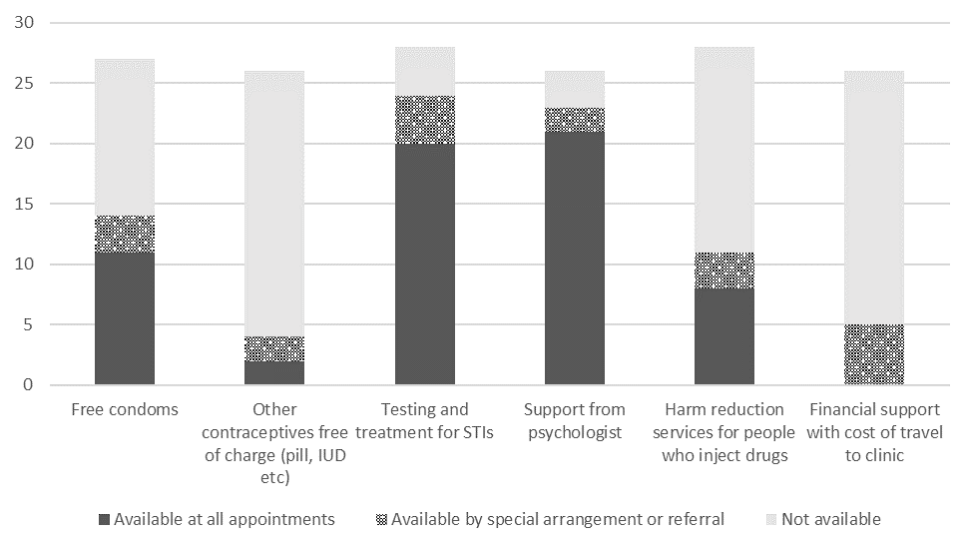
Of 24 regional and 4 municipal centres in Ukraine, the number with each support service available. Bars differ in height due to missing data.

Figure 2 shows the proportion of centres surveyed offering each of six types of service/support in HIV care. Condoms were freely available at half of the centres and other contraceptives at four centres. Testing and treatment for sexually transmitted infections was quite widely available. This was also the case for psychological support; however, the three centres without psychologist support were in regions with some of the highest numbers of young people in HIV care (collectively 7287 of the 13286 10–24 year olds in HIV care nationally). Out of 26 centres answering the question, 17 reported offering a support group, and at six of these 17 centres a group was provided specifically for young people. Eleven centres had harm reduction services available to PWID routinely ( *n*=8) or by referral ( *n*=3). Regarding contributions towards the cost of HIV care in adult services, all 28 centres indicated that ART and general HIV care were free-of-charge, but seven centres indicated patient contributions to costs associated with blood tests ( *n*=5), vaccinations ( *n*=4) and/or general equipment/supplies ( *n*=4). Only five centres offered financial support for travel costs to attend appointments.

Overall, 25/28 centres provided appointment reminders for patients, mainly by phone call (23/25); only one centre reported using SMS and none used email. Twelve centres reported making contact with all patients who missed one follow-up, while 16 centres indicated a selective policy, mostly targeting those on ART ( *n*=14), children or young people in paediatric services ( *n*=15), and/or patients of heightened clinical concern – e.g. those who were pregnant, with low CD4 count, a recently changed ART regimen or preparing to start ART. Patients missing follow-up were most commonly contacted by a doctor (21/28) or nurse (24/28) at the HIV/AIDS centre, a social worker (20/28), a local doctor (13/28) or in one case a psychologist.

## Discussion

Results from this national survey show that of around 13,300 10–24 year olds in HIV care in Ukraine at the beginning of 2016, around 86% (11,400) were aged 20–24 years, with 72% of this age group having sexually-acquired HIV infection. Smaller sub-groups include over 3000 young PWID and increasing numbers of PHIV youth entering adult care. Our survey results indicated that 759 19–24 year olds were newly registered for HIV care in 2015. It is not possible to determine what proportion of all new diagnoses these young people represented (and therefore the proportion linked to care), because national figures are not disaggregated by age group. However, 26% of diagnosed individuals have never been linked to HIV care ^1^.

As expected, young people aged ≤18 years were predominantly cared for in paediatric services. The number of young people transitioning from paediatric to adult care was fairly small – 97 in 2015 – but set to increase year on year. Although transition typically occurs at around 18 years, a range of policies were in place nationally, with some flexibility regarding timing of transition at 17 of 28 centres. This allowed clinicians to be responsive to young people’s developmental maturity and need for continuity of care, which may be important to their feelings of preparedness for transfer and trust in their healthcare provider ^21^. Joint appointments with the paediatrician and adult doctor were part of transition processes alongside psychosocial support in most centres. The close involvement of paediatric and adult staff during transition is recommended in USA guidelines ^10^ and may help young people to overcome barriers to transition which relate to fears of changing relationships with healthcare providers and new systems in adult services ^22^. However, the evidence base for different transition models in improving outcomes is currently lacking. The recent decentralisation of HIV services for adults in Ukraine means that transfer to adult care may be increasingly accompanied by a change in location of care as well as provider; the potential impact of this on retention needs to be examined, along with implications for models of transition support.

Most young people with horizontally-acquired HIV <18 years nationwide start their HIV care in paediatric services. Paediatricians may be better equipped to support adolescents in HIV care and treatment programmes through a greater understanding of adolescent development and behaviour ^23^. However, given the usual age of transfer of 18 years, these young people are likely to lack continuity in their healthcare provider during their first years of HIV care and will navigate transition to adult care with less established relationships with paediatric healthcare providers than their PHIV counterparts. Young people with horizontally-acquired HIV may experience complex barriers to care related, for example, stigma and concerns around confidentiality, which are reflected in longer delays linking to HIV services than is average for older adults ^24^.

The WHO recommends co-location of HIV care with other health services relevant for key populations (e.g. sexual and reproductive health, drug dependence services) ^25^ to improve accessibility of services, which is a key component of youth friendly healthcare. We found uneven availability of services integrated with HIV care at the regional and municipal HIV/AIDS centres included in our survey. Only around half of centres offered free condoms. Other sexually transmitted infections are prevalent ^26^ and condoms are also a main method of family planning in this population ^27^, reflected in the low availability of other forms of contraception as part of HIV care. The 14 centres with free condoms available were in regions with larger caseloads (collectively accounting for 75% of 10–24 year olds in HIV care). Similarly, harm reduction services for PWID were available at 11 centres, located in regions with 86% of young PWID in HIV care nationally, indicating some targeting of services. However, services may not be available to all of the young PLHIV in these regions; for example, harm reduction services were available at only one of the three centres surveyed in each of Kiev and Dnepropetrovsk regions.

Financial support towards transport costs was not available at most HIV centres surveyed. Transport costs are a key potential structural barrier to healthcare, sometimes addressed by clinicians on an informal basis in Ukraine (personal communication, Galyna Kyselyova), and may be compounded by informal costs for some aspects of healthcare (e.g. blood tests). Decentralisation of ART provision may minimise transport-related barriers for young people entering adult HIV care. Routine HIV care was available at almost all centres without an appointment, however only five centres offered services during the evenings or weekends. Availability of flexible care at times which minimise the need to miss school, college or work is an important aspect of age-appropriate care as defined by youth ^21^ and evening clinics have been associated with better retention in care among HIV-positive young people across 12 sites in the USA ^28^. Over half of centres reported a selective policy with regards contact with patients who missed an appointment, with 15/16 of these centres indicating that they would target those in paediatric services. The time of transition to adult care may therefore coincide with diminishing support and follow-up, which is of concern given evidence from other settings of challenges retaining adolescents and young adults in care ^12, 29^. Use of mobile technologies was almost completely absent, but may be a useful means to improve ART adherence and support in HIV care for adolescents ^30^.

Survey results indicated that two-thirds of 10–18 year olds in paediatric HIV care were in just five of the 24 regions, with a similar pattern in 19–24 year olds, reflecting the concentration of the epidemic in the South and East of the country, including some regions also affected by conflict and disruption of HIV and related services in recent years ^31^. While services targeted to these regions will achieve the greatest coverage, different support may be needed in those regions with lower caseloads, tailored to the more limited experience in the clinical and psycho-social care of PHIV children and adolescents (for example, in disclosing to PHIV young people their HIV status) and fewer peer support opportunities.

Peer support opportunities for PHIV young people will increase in coming years as this group reach adolescence and young adulthood in greater numbers, and youth-oriented initiatives (such as those led by community-based organisation Teenergizer) are needed to shape support for the needs of this group alongside youth with horizontally-acquired HIV. The experiences of and barriers to HIV care for all young people need to be explored in the context of Ukraine’s healthcare reforms towards more patient-centred services, and we recently undertook a survey of these groups of young people at two HIV/AIDS centres which investigated these topics, with work ongoing in this area. Importantly, the first cohort of PHIV young people reaching adulthood have different clinical and social characteristics to younger groups – for example, are more likely to be in extended family (vs parental) care – with implications for the level and type of support they may need as they reach adulthood.

There are a number of limitations to this work. We estimated that up to 43 young people may have been double-counted due to simultaneous registration with paediatric and adult services in one region, but could not estimate the number counted by ≥1 region (e.g. due to internal migration or living with extended family members). Figures from the Russian-occupied territories are incomplete. We did not disaggregate young people with sexually-acquired HIV infection into men who have sex with men (MSM) and those with heterosexually-acquired infection, due to the particular vulnerability of MSM in Ukraine which is linked with their under-reporting and misclassification in official figures ^32^, and mode of HIV acquisition may be misclassified in other ways (for example due to late diagnosis of a PHIV young person). Although the number of young people in HIV care in each region gives a complete national picture, we could not accurately estimate their coverage with the services provided at the 28 regional and municipal HIV/AIDS centres included in this survey, because ART and HIV care have been decentralised to smaller local clinics in recent years; services are likely to be less readily available at smaller clinics, particularly for key populations.

## Conclusions

There is substantial heterogeneity in young PLHIV in Ukraine by geography, age and mode of HIV acquisition, and in services at regional HIV/AIDS centres. The number of PHIV young people requiring care during adolescence and young adulthood will continue to increase. Uneven availability of integrated services, financial barriers and lack of evening or weekend services may pose structural barriers to HIV care for young people regardless of mode of HIV acquisition and further work is needed to understand these. The results of this study underscore the importance of including services for adolescents in Ukraine’s comprehensive unified Clinical Protocol on HIV/AIDS, with care models focussed on sustainability, effectiveness and scale up of decentralization of services in the context of wider healthcare reforms in Ukraine.

## Data availability

### Underlying data

Open Science Framework: Young people in HIV care in Ukraine: a national survey on characteristics and service provision. https://doi.org/10.17605/OSF.IO/XS7ZE ^33^.

This project contains the following underlying data:

- Ukraine HIV centre survey data.csv
- Ukraine HIV centre survey data dictionary.csv

### Extended data

Open Science Framework: Young people in HIV care in Ukraine: a national survey on characteristics and service provision. https://doi.org/10.17605/OSF.IO/XS7ZE ^33^.

This project contains the following extended data:

- Final policy survey_English.docx
- Final policy survey_Russian.docx
