# Supplementary material for: Young people in HIV care in Ukraine: a national survey on characteristics and service provision
Source: F1000Res. 2019 May 14;8:323. Originally published 2019 Mar 22. [Version 2] doi: 10.12688/f1000research.18573.2 (PMC6498744; doi:10.12688/f1000research.18573.2)
Supplement: Peer review history

# Peer review: Reviewer response for version 1

**DOI:** 10.5256/f1000research.20330.r47442

**Published:** 2019-05-02

**Authors:** Qiang Xia

**Affiliations:** 1 Division of Disease Control, New York City Department of Health and Mental Hygiene, New York, NY, USA

The authors describe the findings from a national survey of 28 HIV/AIDS centres in Ukraine on the characteristics of young people (10-24 year olds) living with HIV (PLHIV), the policies around the transition from paediatric to adult care, and services provided at these HIV/AIDS centres for young PLHIV. Overall, the methodology is sound, and the data are clearly presented.

Abstract

 The writing could be tightened, especially in the results section, where only major findings should be presented.

Keywords

 Please limit the number of keywords. The authors may consider removing, “injecting drug use,” “reproductive health,” and “harm reduction.”

Abbreviations

 Please make the abbreviations consistent with those in the text, “PHIV, perinatally HIV-infected” vs. “patients with perinatal HIV (PHIV)” in the text, and “BHIV, behaviourally HIV-infected” vs. “behaviourally-acquired HIV (BHIV)” in the text.

Methods

 Please make it clear that there is a total of 28 HIV/AIDS centres, and all of them were included in the survey.

Results

 “Out of 26 centres, 17 reported offering a support group.” Why is it 26 centres not 28 centres? If two centres did not respond to this specific question, state it clearly in the sentence.

 Table 2

 It is not informative to compare types of transition support between centres with no transfers in 2015 and centres with transfers in 2015. If the authors like, they may make comparisons between 24 regional centres and four municipal centres, or by region (e.g., East and West), the size of the patient population, or other characteristics.

 Figure 2

 The bars are not at the same height, because of missing data. The authors may consider adding a category, and showing the data in four categories: 1) service available at all appointments, 2) service available by special arrangement or referral, 3) service not available, and 4) data not available.

Discussion

 “However, 26% of diagnosed individuals overall are currently unlinked to HIV care.” The meaning of the word, “unlinked,” is not clear. If the authors are talking about linkage to care, the sentence should be, “26% of diagnosed individuals overall have never been linked to HIV care.” If the authors are talking about retention in care, the sentence should be, “26% of diagnosed individuals overall are currently not retained in HIV care.”

 The analysis unit of the study is HIV/AIDS centre, e.g., the number and proportion of HIV/AIDS centres with STI testing and treatment services. The analysis is valid, but readers and policy makers are also interested, if not more interested, in the analysis in which patient is the analysis unit, e.g., the number and proportion of patients with access to STI testing and treatment services at HIV/AIDS centres. The authors may consider adding the analysis with patient as the analysis unit in the Results, or listing lack of such an analysis as a limitation in the Discussion.

I have read this submission. I believe that I have an appropriate level of expertise to confirm that it is of an acceptable scientific standard.

# Response

**Published:** 2019-05-02

**Authors:** Heather Bailey

**Affiliations:** University College London, UK

Thank you for the helpful comments.

 We have made some clarifications in the methods and results as suggested.

 In table 2, we made the decision to compare support services at centres with recent transfers and those without to identify any differences in reports of support that may exist in policy vs practice.

 Figure 2 – we have added a footnote to explain that bars differ in height due to missing data.

 Discussion – thank you for the point about the term ‘unlinked’ – in this context we meant that 26% have never been linked to care and have made this edit.

 We are not able to comment on the coverage of individuals with STI testing and treatment (and other) services because of the decentralisation of ART and HIV services to smaller local clinics in recent years. The survey could tell us the total number of young people in each region and availability of each type of support at the regional/municipal centre, but not the precise coverage of young people with these services as part of their HIV care (last paragraph of the discussion).

# Peer review: Reviewer response for version 1

**DOI:** 10.5256/f1000research.20330.r46108

**Published:** 2019-04-16

**Authors:** Lee Fairlie

**Affiliations:** 1 Wits Reproductive Health & HIV Institute, University of the Witwatersrand, Johannesburg, South Africa

Thank you for the opportunity to review this manuscript. This is a well written manuscript describing the population of adolescents and young people living with HIV in the Ukraine, based on a National survey. This manuscript highlights specific characteristics of this population and it is very interesting to see how this data may differ from other parts of the world for example sub Saharan Africa, where the characteristics of PLHIV in this age group is quite different. 

 A few suggestions which could strengthen the manuscript are included below. These are for minor consideration by the authors:

- Consider using vertical transmission and horizontal transmission instead of "behaviourally-acquired" as this term carries substantial stigma. 

Introduction:

- 3rd paragraph, last line, it is reported that 2/3 of PLHIV are on treatment. It is worth elaborating on this and possible reasons in the test and treat era. 

Results:

- In the 2nd paragraph, where age ranges and medians are given, suggest adding IQR, as the range is so vast that this data is difficult to interpret. 
- Suggest adding whether any clinical criteria were considered when transition was decided for example VL suppression, clinical stability (no opportunistic infections, malignancies), pregnancy etc. 
- Under "Support services and follow-up...." separate and discuss separately STIs and psychosocial support, as these are obviously quite different needs and both important. 
- It is really interesting to see data on the mode of HIV acquisition **—**is this information routinely recorded in the notes? A limitation may be that this data may not be accurate and there may be situations where it is difficult to ascertain, for example, if maternal status is unknown or +, a young person who presents late for the first time may have acquired HIV vertically or horizontally.  

Discussion:

- Suggest adding the above to the discussion.
- In the financial support paragraph, suggest also discussing differentiated models of care and also Youth Care Clubs or similar models where less facility-based contact is required. 
- Also consider adding some text regarding the differences between this cohort and others, for example, in SSA .

I have read this submission. I believe that I have an appropriate level of expertise to confirm that it is of an acceptable scientific standard.

# Response

**Published:** 2019-05-02

**Authors:** Heather Bailey

**Affiliations:** University College London, UK

Thank you for the helpful comments.

 We have removed “behaviourally-acquired HIV” as a term and replaced with horizontally-acquired.

 We have edited the line about 2/3 of PLHIV being on treatment to specify that this was in 2016 (it was similar in 2017, more recent data not yet available).

 Results – we have added IQR as suggested. Clinical criteria for postponing transition were captured with the option “young person is in poor health” which was selected by one centre. The section about STIs and psychosocial support has been edited to separate these out. Data on mode of HIV acquisition is routinely reported, however there is evidence of misclassification of MSM as having heterosexually-acquired HIV infection which we describe in the limitations section – we have also added some text to say that “mode of HIV acquisition may be misclassified in other ways (for example due to late diagnosis of a PHIV young person).”

 We agree that decentralisation of care is an important point for overcoming financial barriers to travelling to the regional/municipal HIV/AIDS centre and mention this.
